# Denosumab in pediatric patients with fibrous dysplasia/McCune-Albright syndrome: a single-center, open-labeled study

**DOI:** 10.3389/fendo.2026.1762370

**Published:** 2026-02-18

**Authors:** Quanshi Ouyang, Ruizhi Jiajue, Ruotong Zhou, Wenting Qi, Xiang Li, Lijia Cui, Yue Chi, Qianqian Pang, Yiyi Gong, Wei Liu, Yan Jiang, Ou Wang, Mei Li, Xiaoping Xing, Weibo Xia

**Affiliations:** 1 Department of Endocrinology, Key Laboratory of Endocrinology, State Key Laboratory of Complex Severe and Rare Diseases, National Commission of Health, Peking Union Medical College Hospital, Chinese Academy of Medical Sciences, Beijing, China; 2 Department of Internal Medicine, Plastic Surgery Hospital, Chinese Academy of Medical Sciences and Peking Union Medical College, Beijing, China

**Keywords:** denosumab, fibrous dysplasia, hypercalcemia, McCune-Albright syndrome, pediatric

## Abstract

**Context:**

Fibrous Dysplasia/McCune-Albright Syndrome (FD/MAS) is a rare skeletal disorder frequently manifesting in childhood, often leading to progressive bone lesions, pain and functional impairment. Denosumab as a monoclonal antibody targeting RANKL has emerged as a potential therapeutic option, while its safety and efficacy in pediatric population remains poorly defined.

**Objective:**

Investigate the efficacy and safety of denosumab in pediatric FD/MAS population.

**Design:**

12-month single-arm study.

**Setting:**

Single center study at Peking Union Medical College Hospital.

**Patients:**

FD/MAS patients under 18.

**Interventions:**

Denosumab 1mg/kg with a maximum dosage of 60mg every 3 months for 12-month follow-up.

**Main outcome measures:**

FD-related bone pain, bone turnover markers, ^99m^Tc-MDP bone scintigraphy, bone mineral density.

**Results:**

In 5 pediatric FD/MAS patients treated with denosumab, significant clinical improvements were observed, including alleviation of FD-associated bone pain, reductions in bone turnover markers, and regression of FD lesions. Alkaline phosphatase levels dropped to an average of 41.8% of baseline, accompanied by concurrent reductions in C-terminal telopeptide and type 1 N-terminal pro-peptide levels. Adverse events particularly hypercalcemia following treatment cessation occurred in 2 of the 5 patients, indicating a relatively high incidence of rebound hypercalcemia and highlighting the need for careful monitoring and cautious use of denosumab in pediatric patients.

**Conclusions:**

Denosumab appears to be effective in pediatric FD/MAS patients, however safety concerns remain. Comprehensive pre-treatment evaluation and close monitoring throughout the treatment course are essential. Further prospective studies and randomized controlled trials are needed to determine optimal dosing strategies and establish long-term safety in this population.

## Introduction

Fibrous dysplasia (FD) is a congenital disease caused by somatic mutations in the *GNAS* gene, which encodes G-protein α-subunit (Gαs) in the osteoblastic cell lineage. The underlying molecular mechanism of the disease is hyperactivation of adenyl cyclase *in vivo* and subsequent excessive production of cAMP, disrupting normal osteoblast differentiation and osteoclast function (1, 2). Patients with FD commonly experience bone pain, deformities, and fractures due to elevated bone turnover and impaired bone microstructure. FD sometimes manifested as a component of McCune-Albright Syndrome (MAS), alongside characteristic café-au-lait skin macules and hyperfunctioning endocrinopathies, including precocious puberty, hyperthyroidism, and acromegaly. Both FD and MAS are associated with severely reduced quality of life, underscoring the need for effective medical management. The prevalence of FD/MAS was estimated at 61.0 per 1,000,000 individuals in Denmark in 2018 (3). In developing countries, the true prevalence may be underestimated due to higher birth rates and limited availability of prenatal diagnostic methods. These factors emphasize the unmet need for optimized therapeutic and follow-up strategies for patients with FD/MAS.

In pediatric patients, the need for effective therapeutic strategies is particularly critical. FD/MAS often manifests during childhood and progresses actively throughout adolescence, leading to the expansion of skeletal lesions and worsening bone pain. These clinical observations underscore the importance of early and proactive management during the pediatric stage. However, there remains a lack of systematic studies evaluating the safety and efficacy of available treatments in this population.

Bisphosphonates have been used for several decades as the standard treatment for FD/MAS, and have demonstrated efficacy in alleviating bone pain in a significant proportion of patients (4–6). However, as anti-resorptive agents that primarily inhibit osteoclast attachment and activity on the bone surface, bisphosphonates are mechanistically limited in their capacity to reduce FD lesions. Data indicate that bisphosphonates have limited efficacy in reducing skeletal burden and, more specifically, demonstrate no benefit in controlling craniofacial lesions, as shown in a large cohort of 31 patients with craniofacial FD treated with bisphosphonates (7). Particularly in pediatric patients with craniofacial involvement, given the rapid skeletal growth and modeling drift, static anatomical lesions can rapidly progress to cause deformity and subsequent complications, including neural compression and impaired somatic growth; therefore, effective lesion control is urgently needed to prevent disease progression. In this context, bisphosphonate therapy often falls short of achieving adequate therapeutic efficacy. Although zoledronate and pamidronate have demonstrated substantial efficacy in alleviating FD-related bone pain, often resulting in near-complete pain resolution (1), Florenzano et al. reported in a 2019 study that one year of zoledronate treatment did not result in a reduction in SBS, with a mean increase of 3.1 observed among patients younger than 15 years. BTMs demonstrated heterogeneous responses during treatment. ALP levels did not differ from those in untreated pediatric patients, whereas β-CTX and P1NP decreased by only approximately 18% on average (8, 9).

More recently, denosumab has shown promising therapeutic effects in patients with FD/MAS, especially in those who were refractory to bisphosphonate therapy (10, 11). Several case reports have described reductions in skeletal disease burden following denosumab treatment, including suppressed bone turnover, reduced lesion activity on bone scintigraphy, and even diminished lesion. These findings support the potential role of denosumab as a preferable treatment option for FD/MAS (12, 13). Based on these reports and available clinical data in adult patients, we hypothesized that denosumab might confer superior efficacy compared with conventional bisphosphonate therapy in pediatric patients with FD/MAS, and we designed this study to test this hypothesis. Meanwhile, given the frequent reports of post-injection hypocalcemia and rebound hypercalcemia during drug cessation or dose adjustment, the safety profile of denosumab represents another critical endpoint requiring careful evaluation, especially in pediatric patients.

In our study, we recruited 5 FD/MAS patients under the age of 18 and treated them with a standard denosumab regimen. We aimed to evaluate the drug’s efficacy in early disease control, focusing on outcomes such as bone pain relief, suppression of bone turnover, and reduction in lesion size and radioactivity uptake. Importantly, we designed strict monitoring of adverse events during and after follow-up, to ensure the safety of treatment in this special pediatric population, and provided inferable instructions for their prevention and management.

## Materials and methods

### Study design

This was a single-center, open-label, single-arm study designed to evaluate the effects of denosumab treatment in pediatric FD/MAS patients. Patients aged 18 years or younger were recruited from the Department of Endocrinology at Peking Union Medical College Hospital (PUMCH). Denosumab (Prolia, Amgen Inc, USA) was administered via subcutaneous injection at a dosage of 1mg/kg with a maximum dosage of 60mg every 3 months (14) according to previous clinical experience. Calcium supplementation was provided at 500mg/day for patients aged 6 to 12 years old, and 1000mg/day for those older than 12 years. Additionally, all patients received Vitamin D3 at a daily dose of 600–1000 IU, along with calcitriol at 20-30ng/kg daily for up to 4 days post-injection to maintain serum calcium levels. The study protocol was approved by the Scientific Ethics Committee of PUMCH (approval code: ZS-3191), and written informed consents were obtained from the legal guardians of all participants prior to enrollment.

### Safety measures

Given the well-documented risk of adverse events associated with denosumab therapy, particularly acute hypocalcemia following administration, and hypercalcemia during dosage tapering or discontinuation in pediatric patients, we implemented safety monitoring protocol. During the follow-up period and for up to 6 months afterward, patients were required to have their blood calcium and phosphate levels tested monthly. If hypo- or hypercalcemia was detected, patients were instructed to return to PUMCH for prompt medical management. For hypocalcemia, the protocol involved adjusting oral calcium and calcitriol dosages, with intravenous calcium supplementation administered to symptomatic patients as necessary. In the event of hypercalcemia, calcium and calcitriol supplements were immediately discontinued. Patients with moderate-to-severe (serum calcium ≥3.0 mmol/L) or symptomatic hypercalcemia were treated with calcitonin, followed by either re-administration of denosumab or a transition to zoledronate, depending on the individual clinical context. Even after the formal follow-up concluded, patients continued with ongoing therapeutic strategies, which included either denosumab therapy or a transition to sequential zoledronate administration, along with follow-up visits every 3 months at PUMCH. The dosing and frequency of continuous therapy were personalized based on disease activity and individual patient profiles.

### Patient recruitment

Eligibility criteria included pediatric patients with a confirmed diagnosis of FD/MAS. All patients were required to have baseline serum alkaline phosphatase (ALP) levels above the upper limit of normal, and to exhibit other clinical signs of active disease, such as bone pain, skeletal deformities, or symptoms of neural compression. For patients diagnosed with MAS, at least one characteristic feature was required, including café-au-lait macules, hyperthyroidism, precocious puberty, or growth hormone (GH)-secreting pituitary adenoma with evidence of GH hypersecretion. Exclusion criteria included a prior history of denosumab treatment or any contraindications to its use, such as hypocalcemia or a history of osteonecrosis of the jaw. Patients with comorbidities known to affect bone metabolism, such as primary hyperparathyroidism, immobilization-related bone loss, or severe hepatic or renal dysfunction were also excluded. In addition, those receiving medications that significantly impact bone metabolism, including glucocorticoids, were ineligible. The recruitment period spanned from June 2021 to July 2023. Patients who experienced serious adverse events during follow-up or whom voluntarily withdrawn consent were excluded from final analysis. A detailed flowchart outlining the inclusion and exclusion process is provided in **Supplementary Figures S1**, **S2**. Finally, 5 eligible patients were enrolled in the study in accordance with the predefined criteria.

### Study assessment

The primary endpoints of this study included bone pain assessed using the visual analogue scale (VAS), skeletal burden score (SBS) derived from ^99m^Tc-methylene diphosphonate (MDP) bone scintigraphy, and serum ALP levels. Serum ALP was used as a surrogate marker of overall bone turnover. SBS and VAS pain scores reflect the extent of skeletal involvement and the severity of disease related impairment in quality of life. Secondary endpoints included serum levels of β-type I collagen carboxyterminal peptide (β-CTX) and N-terminal pro-peptide of type I pre-collagen (P1NP), and bone mineral density (BMD) at femoral neck, lumbar spine and total hip. Trends of β-CTX and P1NP changes provided supportive evidence for changes in bone remodeling activity, while longitudinal assessment of BMD allowed evaluation of physiological bone mass accrual and potential effects of anti-resorptive therapy on skeletal development in pediatric patients. Fasting serum samples were collected and analyzed every 3 months over a 1-year follow-up period, during which bone pain was assessed concurrently. Bone scintigraphy and SBS calculation were performed at baseline and at the end of the follow-up period. All serum biochemical analysis were conducted at the central clinical laboratory of PUMCH. ALP, calcium and phosphate levels were measured using automated analyzers (ADVIA 1800, Siemens, Germany), while total 25-hydroxyvitamin D (25OHD) and intact parathyroid hormone (PTH) levels were determined using an automated electro-chemiluminescence system (E170, Roche Diagnostics, Switzerland). BMDs were tested in lumbar spine (LS), femoral neck (FN) and total hip (TH) with dual-energy X-ray absorptiometry (DXA, Lunar Prodigy Advance, GE Healthcare, USA). Bone scintigraphy was performed in the PUMCH Department of Nuclear Medicine, using 25mCi of ^99m^Tc-MDP as the radiotracer. The SBS was calculated according to the method proposed by Collins et al. (15), which reflects the extent of skeletal involvement and is positively correlated with the number of affected bones. Final SBS scores were determined through independent back-to-back assessments. VAS pain scores were assessed at 3-month intervals at our center, in accordance with the scheduled follow-up visits.

### Statistical analysis

Statistical analyses and graphing were conducted using GraphPad Prism 9.5.0 for MacOS (Boston, Massachusetts). Descriptive analysis was performed based on the collected data and reference ranges of Chinese pediatric population.

## Results

### Baseline characteristics

A total of 6 pediatric patients with a confirmed diagnosis of FD/MAS were enrolled at our center and completed the 12-month treatment and follow-up protocol, 1 voluntarily withdrew consent, and 5 pediatric patients were available for inclusion in the final analysis. Baseline serum biochemical markers and clinical characteristics of the 5 FD/MAS patients are summarized in **Tables 1**, **2**. All patients presented with polyostotic FD involving the craniofacial bones. 4 of the 5 patients exhibited one or more endocrinopathies consistent with the diagnosis of MAS. 2 out of 4 had precocious puberty, 3 out of 4 had hyperthyroidism, 3 out of 4 had GH-secreting pituitary adenoma, and 2 out of 4 had hypophosphatemia.

**Table 1:** Baseline levels of biochemical markers.

Biochemical markers	Mean (min, max)	Reference
Ca (mmol/L)	2.40(2.38-2.45)	2.25-2.75
P (mmol/L)	1.18(1.06-1.21)	1.10-1.30
T-25OHD (ng/mL)	23.3(21.4-29.1)	8.0-30.5
PTH (ng/L)	35.4(27.7-39.0)	12.0-88.0
GGT (U/L)	18(15-23)	7-45
ALP (U/L)		
Absolute value	643(203-876)	42-390(0-15yr)
		52-171(16-18yr)
Divided by ULN	1.78(1.42-2.02)	<1
β-CTX (ng/mL)	3.12(1.12-5.13)	0.21-0.44
P1NP (ng/mL)	1158(260-2400)	15.1-58.6
BMD (g/cm^3^)		
FN BMD	0.847(0.748-1.133)	
LS BMD	0.864(0.502-1.123)	
TH BMD	0.851(0.686-1.072)	

**Table 2:** Baseline characteristics of all 5 participants.

No.	Sex/Age	MAS/PFD	Affected bones	Bone pain VAS-score	SBS-score	ALP (U/L)	β-CTX (ng/mL)	P1NP (ng/mL)	Café-au-lait macule	Precocious puberty	Hyper-thyroidism	GH-secreting pituitary adenoma	Hypo- phosphatemia
P1	M/8	MAS	Skull, right pelvis, bilateral upper extremities, right lower extremity	8	30.41	789	2.01	>2400	+	–	+	+	–
P2	F/4	MAS	Skull, bilateral femur	4	18.51	731	1.99	1329	+	+	+	–	+
P3	M/6	MAS	Skull, bilateral pelvis, bilateral upper and lower extremities	0	49.26	585	3.59	1298	+	–	+	+	+
P4	M/16	PFD	Skull, bilateral humerus	0	12.08	203	1.19	260	–	–	–	–	–
P5	F/8	MAS	Skull	3	11.60	876	5.13	502	+	+	–	+	–

At baseline, all patients had serum ALP levels exceeding the age-specific upper limit of normal (ULN). Additionally, BTMs reflective of both bone formation and resorption, specifically β-CTX and P1NP were markedly elevated. Serum calcium, phosphate, total 25OHD, and parathyroid hormone (PTH), were within normal reference ranges. Gamma-glutamyl transpeptidase (GGT) levels were also within the normal range for all participants, suggesting preserved hepatic function and supporting the validity of using serum ALP as a reliable marker of bone turnover in this cohort.

None of the participants had received prior treatment with denosumab or bisphosphonates before study enrollment.

### Alleviation of bone pain

**Figure 1:**
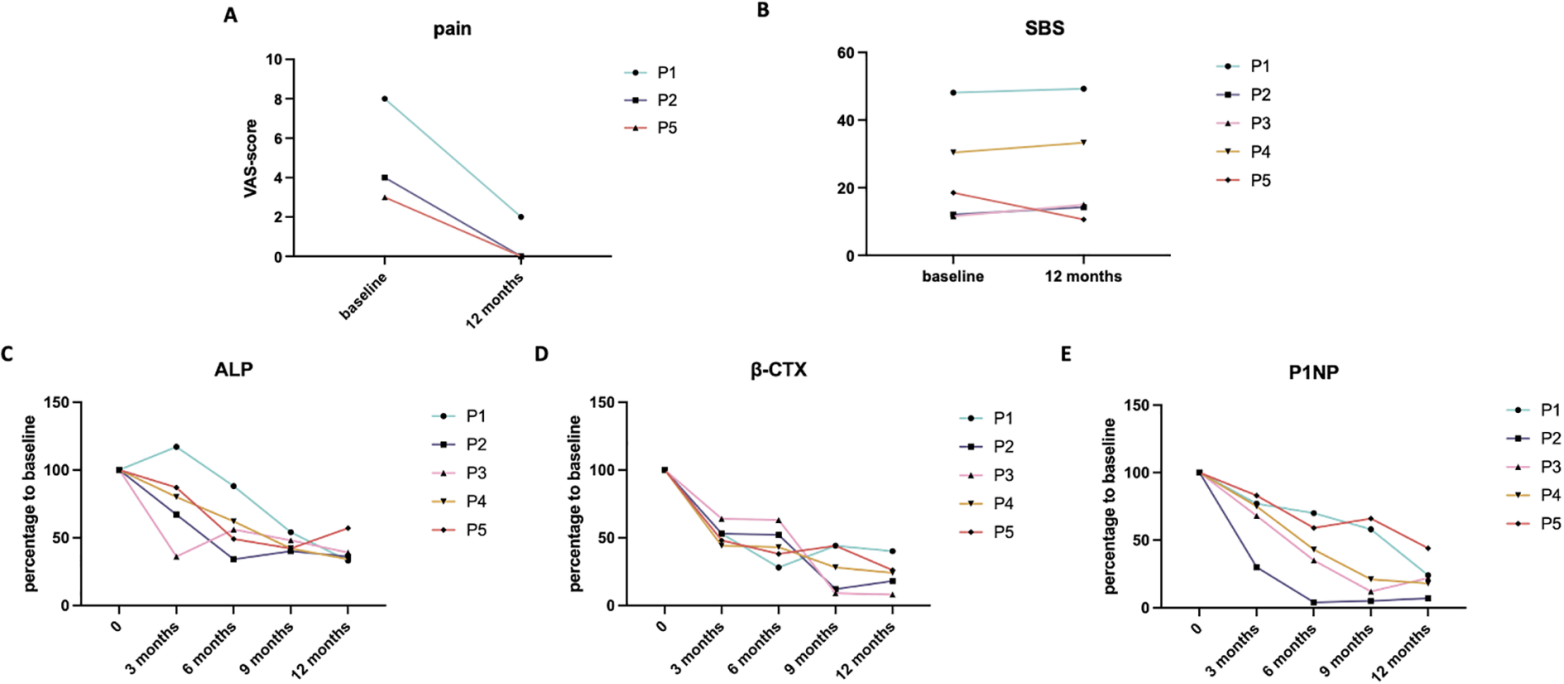
Longitudinal trends of changes in FD-associated bone pain, SBS, and BTMs. **(A)** Reduction of FD-associated bone pain after follow-up in 3 patients (P1, P2, P5) with baseline bone pain. **(B)** Changes in SBS during follow-up. **(C–E)** Percentage changes of BTMs relative to baseline during follow-up.

At the time of recruitment, 3 out of 5 patients reported varying degrees of FD-associated bone pain., affecting regions involving the skull, ankle, femur, and tibia. Following treatment with denosumab, a marked reduction in bone pain was observed in these patients (**Figure 1A**). Importantly, no new onset of bone pain occurred in the two patients who were asymptomatic at baseline.

### Bone turnover suppression

All 5 patients presented with elevated serum ALP levels at baseline, with an average of 1.77 times the ULN. As shown in **Figure 1C**, longitudinal monitoring revealed consistent reductions in ALP across all participants during follow-up, with individuals decreased to 33%, 46%, 39%, 34%, and 57% of baseline values. By the end of the 12-month follow-up, the average ALP level had declined to 0.76 times the ULN, with 4 out of 5 patients achieving values within the normal reference range.

Similarly, serum levels of β-CTX and P1NP demonstrated parallel declines over the course of treatment, as illustrated in **Figures 1D, E**. At the final follow-up, β-CTX levels had decreased to 40%, 18%, 8%, 24%, and 26% of baseline levels across the 5 patients, while P1NP levels had decreased to 24%, 7%, 22%, 18%, and 44% of baseline, respectively.

Meanwhile, despite the significantly suppression of bone turnover, all 5 patients demonstrated gains in BMD (data shown in **Supplementary Figure S3**), and sites tested with BMD were not involved in lesion sites, suggesting that the anti-resorptive effects of denosumab did not adversely affect bone mass accrual in these pediatric patients.

### Reduction of FD lesions

**Figure 2:**
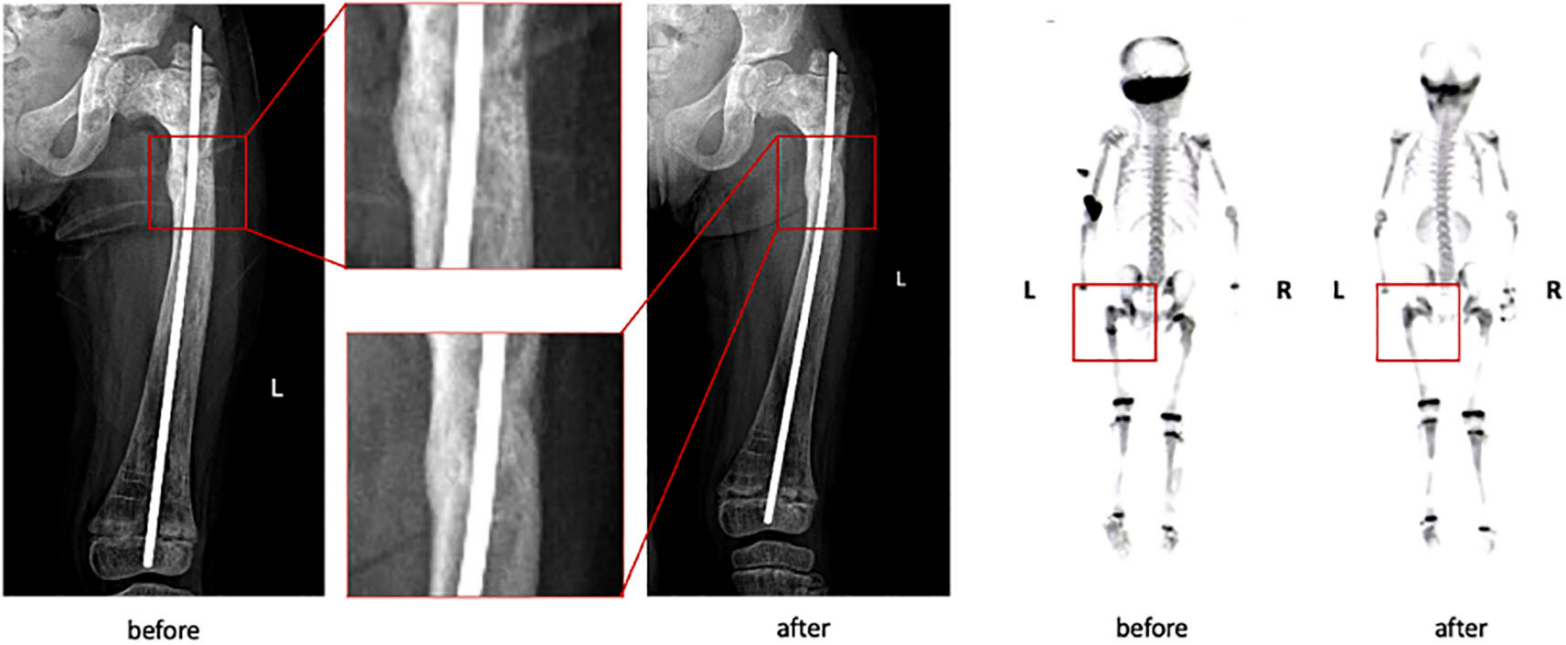
X-ray and bone scintigraphy of P2 before and after follow-up, showing reduction of left femoral and skull base lesion.

All patients were diagnosed with polyostotic FD, with a mean SBS of 24.37 at baseline (range: 11.60–49.26), as assessed by ^99m^Tc bone scintigraphy. By the end of the follow-up period, SBS increased in 4 patients, with a mean increase of 2.39, and patient **P2** demonstrated a notable reduction in SBS, decreasing from 18.51 to 10.64 (**Figure 1B**). This improvement was supported by visibly reduced abnormal radiotracer uptake in the left femur and a decrease in lesion size at the skull base, with X-ray and bone scintigraphy data shown in **Figure 2**. While the SBS remained unchanged in the other 4 patients, focal reductions in radiotracer uptake were observed in several skeletal regions, though these findings are not shown in the figures.

### Denosumab-relevant adverse events

All adverse events occurred during follow-up of all 5 patients are listed in **Table 3**. Among the 5 patients treated with denosumab, transient hypocalcemia was observed in patients **P2** and **P3** following the first injection. The nadir serum calcium levels were 1.74 mmol/L and 1.83 mmol/L, respectively. Both cases were managed effectively with increased supplementation of calcium carbonate (1,200 mg/day) and calcitriol (0.5 µg/day) prior to the second denosumab administration.

**Table 3:** Dosage and adverse events during denosumab medication.

No.	1^st^ course dosage	2^nd^ course dosage	3^rd^ course dosage	4^th^ course dosage	Accumulated dosage	Adverse events
P1	40 mg	40 mg	40 mg	40 mg	160 mg	No prominent adverse event
P2	30 mg	30 mg	30 mg	30 mg	120 mg	Occurrence of **hypercalcemic crisis** after 4^th^-course denosumab injection; occurrence of **hypocalcemia** after 1^st^ -course injection
P3	30 mg	30 mg	30 mg	30 mg	120 mg	Occurrence of **hypercalcemia** after 4^th^-course denosumab injection; occurrence of **hypocalcemia** after 1^st^-course injection
P4	60 mg	60 mg	60 mg	60 mg	240 mg	Occurrence of Epididymal head cyst after 4^th^-course denosumab injection
P5	40 mg	40 mg	40 mg	40 mg	160 mg	No prominent adverse event

Bolded text indicates typical and severe adverse events during denosumab medication.

Conversely, both **P2** and **P3** also experienced hypercalcemia after denosumab cessation, both at the 4^th^ month after the last dosage of denosumab was given. Hypercalcemic crisis occurred in **P2** initially presented with influenza-like symptoms such as fever, poor appetite, followed by nausea, vomiting, and abdominal pain. Upon admission to PUMCH, serum calcium was measured at 4.0 mmol/L. The patient was treated with a combination of intravenous fluids, calcitonin, and a single dose of zoledronate, which successfully reduced the serum calcium level to 2.4 mmol/L. Patient **P3** developed hypercalcemia with emergent tested serum calcium 3.13 mmol/L, management with calcitonin and fluid supplementation led to normalization of calcium levels.

## Discussion

This study represents the first clinical investigation to evaluate the efficacy and safety of denosumab in pediatric patients with FD/MAS. Notably, all enrolled patients presented with polyostotic lesions involving the skull, highlighting the clinical significance of this research in managing early-stage, high-burden FD/MAS in children. The findings demonstrated that denosumab effectively suppressed disease activity, as evidenced by substantial reductions in serum ALP levels and attenuation of lesion size and radiotracer uptake, in parallel with decreases in β-CTX and P1NP levels. Meanwhile, all patients demonstrated increases in BMD during follow-up, as confirmed by DXA assessments performed at baseline and at the end of the follow-up period. These findings indicate that denosumab therapy did not impair calcium deposition or BMD accrual at the femoral neck, lumbar spine and total hip. The assessed sites were free of FD involvement and were therefore excluded from lesion-specific analysis. These results provide valuable insight into potential therapeutic strategies for pediatric FD/MAS patients, a population often characterized by poor prognosis due to high bone turnover, craniofacial involvement, and multiple endocrinopathies associated with MAS.

Historically, bisphosphonates including alendronate, pamidronate and zoledronate have served as the cornerstone of FD/MAS treatment for several decades. These agents have demonstrated efficacy in preserving BMD and reducing BTMs (6). In particular, intravenous administration of pamidronate and zoledronate has been shown to significantly alleviate FD-associated bone pain, emphasizing their utility in pain management (16, 17). However, multiple studies have reported limitations of bisphosphonates, especially in patients with markedly elevated bone turnover, extensive skeletal involvement, and craniofacial lesions (18, 19). This clinical profile is common among pediatric FD/MAS patients, underscoring the pressing need for individualized treatment strategies and age-specific clinical guidelines.

The findings of our study confirm the efficacy of denosumab in the pediatric population, particularly in reducing lesion-specific bone turnover activity and overall skeletal burden. Previous research has identified a positive correlation between RANKL expression and skeletal burden in FD/MAS patients (20). Denosumab, as a humanized monoclonal antibody targeting RANKL, has already demonstrated efficacy in reducing skeletal burden in adult FD patients (12, 13). Combined with our results, these data provide a refreshed general view that denosumab is effective across pediatric and adult age groups. Collectively, this evidence supports denosumab as a promising therapeutic alternative for managing FD in FD/MAS patients. Notably, for pediatric FD/MAS patients who often present with higher disease activity and reduced responsiveness to conventional treatments, denosumab may offer a more effective option when administered under careful clinical supervision.

In our study, denosumab demonstrated remarkable efficacy in controlling FD-associated bone pain, suppressing bone turnover, and reducing SBS in pediatric patients. Among the 3 patients who reported baseline bone pain, all experienced substantial relief, with no recurrence during the follow-up period. Serum ALP levels were reduced to 39 ± 9% of baseline, with 4 of 5 patients achieving values below the upper limit of normal. Based on our center’s experience with bisphosphonate therapy in FD/MAS (4), which typically results in a mean reduction to approximately 69% of baseline, denosumab appeared to demonstrate greater efficacy in suppressing this bone turnover marker. Given that ALP serves as a key marker of overall bone turnover, this reduction suggests effective and sustained suppression of FD-lesion activity. This interpretation is further supported by the marked decreases in serum β-CTX and P1NP levels observed across all patients.

Reduction in lesion radioactivity uptake was also observed in patients during follow-up. One patient demonstrated a decrease in the size of the skull base lesion and near-complete resolution of the FD lesion in the left femur, resulting in a reduction of the SBS score from 18.51 to 10.64. Other patients exhibited varying degrees of decreased radioactivity uptake, although no significant changes in lesion size were noted. These findings may suggest a partial alleviation of disease burden, albeit insufficient to produce a significant reduction in SBS score (12). Similar lesion regression has been reported in other clinical trials investigating denosumab, though with differences in dosage and administration schedules. Notably, most cases documenting lesion regression involved higher denosumab doses (120 mg every three months or at shorter intervals) compared to the 60 mg every 3 months regimen used in our study, implying that higher doses may be more effective in managing FD (12).

Despite the notable therapeutic benefits, adverse events, particularly hypercalcemia were observed, consistent with existing literature. Common adverse effects associated with denosumab include hypocalcemia, osteonecrosis of the jaw, hypercalcemia following drug cessation, rash, and dizziness, with hypercalcemia representing one of the most severe complications (12, 21). The safety of denosumab in pediatric patients with FD/MAS was first noted by Collins et al. in a 2012, who reported severe rebound hypercalcemia following drug cessation in a 9-year-old boy (14). Case reports remain scarce. Although reports on the use of denosumab in pediatric patients with FD/MAS are limited, its safety profile has been more extensively characterized in pediatric patients with osteogenesis imperfecta (22). In this population, hypercalcemia has been commonly reported, with approximately 14.3% of patients progressing to hypercalcemic crisis, which is informative for interpreting the safety concerns in our study. The hypercalcemia observed upon denosumab discontinuation may be explained by rebound osteoclastogenesis mediated by osteomorphs (23), or by rapid resorption of the excessive bone tissue formed during treatment. Patients **P2** and **P3** experienced hypocalcemia prior to the onset of hypercalcemia, indicating an initially adequate osteoclastic suppression by denosumab, followed by an exaggerated rebound in bone resorption when timely follow-up was lacking. The risk of hypercalcemia may be exacerbated by factors such as prolonged drug withdrawal, hypervitaminosis D, immobilization, excessive calcium intake, or hyperthyroidism (21, 24–26), particularly given that all patients received calcium and vitamin D supplementation following denosumab administration. In these 2 cases, hypercalcemia was precipitated by unsupervised calcium supplementation and a lack of adherence to safety monitoring protocols, causing nausea and vomiting, which contributed to dehydration and clinical deterioration, ultimately culminating in a hypercalcemic crisis. This underscores the critical need for strict patient education and rigorous monitoring strategies to ensure patient safety, rather than solely attributing the events to drug mechanisms. These findings underscore the need for strict safety monitoring during denosumab therapy and emphasize the importance of patient adherence to treatment protocols. When follow-up procedures and safety monitoring are rigorously observed, the risk of adverse events during denosumab treatment can be effectively controlled.

Based on our center’s experience in treating FD/MAS, hypercalcemia is the most common adverse event observed during denosumab cessation or interval adjustments. This complication is particularly frequent and severe in pediatric patients due to their relatively high bone metabolic activity. Conventional management strategies for cessation-related hypercalcemia include hydration, administration of calcitonin, and corticosteroid therapy (27). However, a standardized approach for long-term prevention has not yet been established due to insufficient data. Common preventive strategies involve close monitoring of serum calcium, phosphate, and BTMs, along with the use of sequential bisphosphonate therapy following denosumab discontinuation (27, 28). The rationale for sequential bisphosphonate use is to transition from denosumab to a less potent anti-resorptive agent, thereby mitigating the abrupt rebound of bone resorption. In clinical practice, zoledronate is commonly used at a dose of 0.025mg/kg every 3 months, with the first dose given 3 months after denosumab cessation (29). Monthly monitoring of serum markers is recommended for the first 3 months following denosumab cessation or interval adjustment to allow early detection of rebound hypercalcemia. For patients whose serum calcium exceeds the upper limit of normal, an immediate dose of zoledronate 0.025mg/kg is advised. If hypercalcemia persists, administration of denosumab at 0.25mg/kg may be considered (27).

Moreover, recent studies have shown that denosumab administered at a dose of 120mg every 4 weeks can effectively control disease activity in patients with FD/MAS without a high incidence of severe adverse events, although cases of rebound hypercalcemia following treatment discontinuation have been reported (12). We hypothesize that optimization of denosumab dosing strategies and administration intervals may mitigate adverse effects while maximizing therapeutic efficacy; therefore, future prospective studies are warranted to establish safer and more effective treatment protocols for this unique pediatric patient population.

### Study limitations

This study is constrained by a limited sample size, primarily due to the rare prevalence of FD/MAS, the stringent inclusion criteria for pediatric patients, and the cautious selection of treatment regimens for this vulnerable population. In addition, the open-label design and patient-reported VAS pain assessments introduce potential bias, the assessment of bone pain was not blinded and reported by patients to different clinicians on duty. Accordingly, conclusions regarding pain reduction should be interpreted with caution alongside objective markers like SBS and BTMs. The conclusion regarding pain reduction was derived from data from only 3 patients with baseline bone pain out of the 5 patients, thereby limiting the strength of this conclusion. The study also lacked a comparison group treated with conventional bisphosphonates, therefore attributing the observed effects solely and directly to denosumab should be interpreted with caution. To improve the robustness of future research, efforts should focus on establishing larger FD/MAS cohorts and conducting randomized controlled trials.

Regarding the diagnostic criteria, only two of the five patients (P1 and P3) in this cohort underwent GNAS sequencing by NGS of peripheral blood mononuclear cells, and a pathogenic mutation was identified in only one patient (P3). As a result, molecular confirmation was available in a limited proportion of cases. Nevertheless, the diagnosis of FD/MAS in this cohort remains reliable, as it was established based on characteristic clinical, biochemical, and radiological features in accordance with international guidelines. Future advances in more sensitive molecular techniques, such as cell-free DNA–based GNAS sequencing, may further improve diagnostic accuracy in FD/MAS.

In the evaluation of FD lesions, the SBS score offers a useful general overview of skeletal involvement. However, it may not accurately capture lesion dynamics in all cases. Specifically, the SBS score may remain unchanged in patients whose extent of bone involvement appears stable, even when there is a significant reduction in lesion size or radioactivity uptake. Alternative imaging modalities, such as ^18^F-NaF PET/CT, may provide a more precise assessment of FD lesion progression and should be considered in future studies (30).

Finally, extended follow-up is essential for thoroughly evaluating the long-term effects of denosumab treatment in FD/MAS patients, enabling a more comprehensive understanding of its sustained safety and efficacy.

## Conclusion

The findings of our study highlight the efficacy of denosumab in treating pediatric FD/MAS patients. Denosumab demonstrated significant benefits in alleviating FD-associated bone pain, reducing bone turnover, and controlling lesion activity.

Nonetheless, safety considerations must remain a central focus during denosumab therapy, particularly in relation to MAS manifestations, baseline BTMs levels, treatment regimens, and patient adherence. These factors may influence both the risk of severe adverse events and overall treatment effectiveness. Therefore, comprehensive pre-treatment evaluation and close monitoring throughout the treatment course are essential to ensure patient safety. The risk of adverse events during denosumab treatment can be effectively controlled when sticking to strict follow-up procedures and safety monitoring.

## Data Availability

The original contributions presented in the study are included in the article/**Supplementary Material**. Further inquiries can be directed to the corresponding author.
