# Supplementary material for: Denosumab in pediatric patients with fibrous dysplasia/McCune-Albright syndrome: a single-center, open-labeled study
Source: Front Endocrinol (Lausanne). 2026 Feb 18;17:1762370. doi: 10.3389/fendo.2026.1762370 (PMC12956703; doi:10.3389/fendo.2026.1762370)
Supplement: Supplementary file 1 [file DataSheet1.docx]

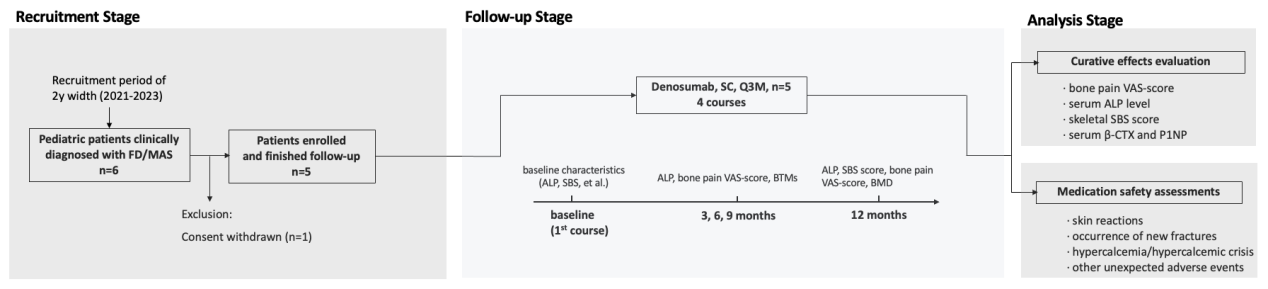


**Supplementary figure 1.** Inclusion/exclusion criteria and follow-up design.


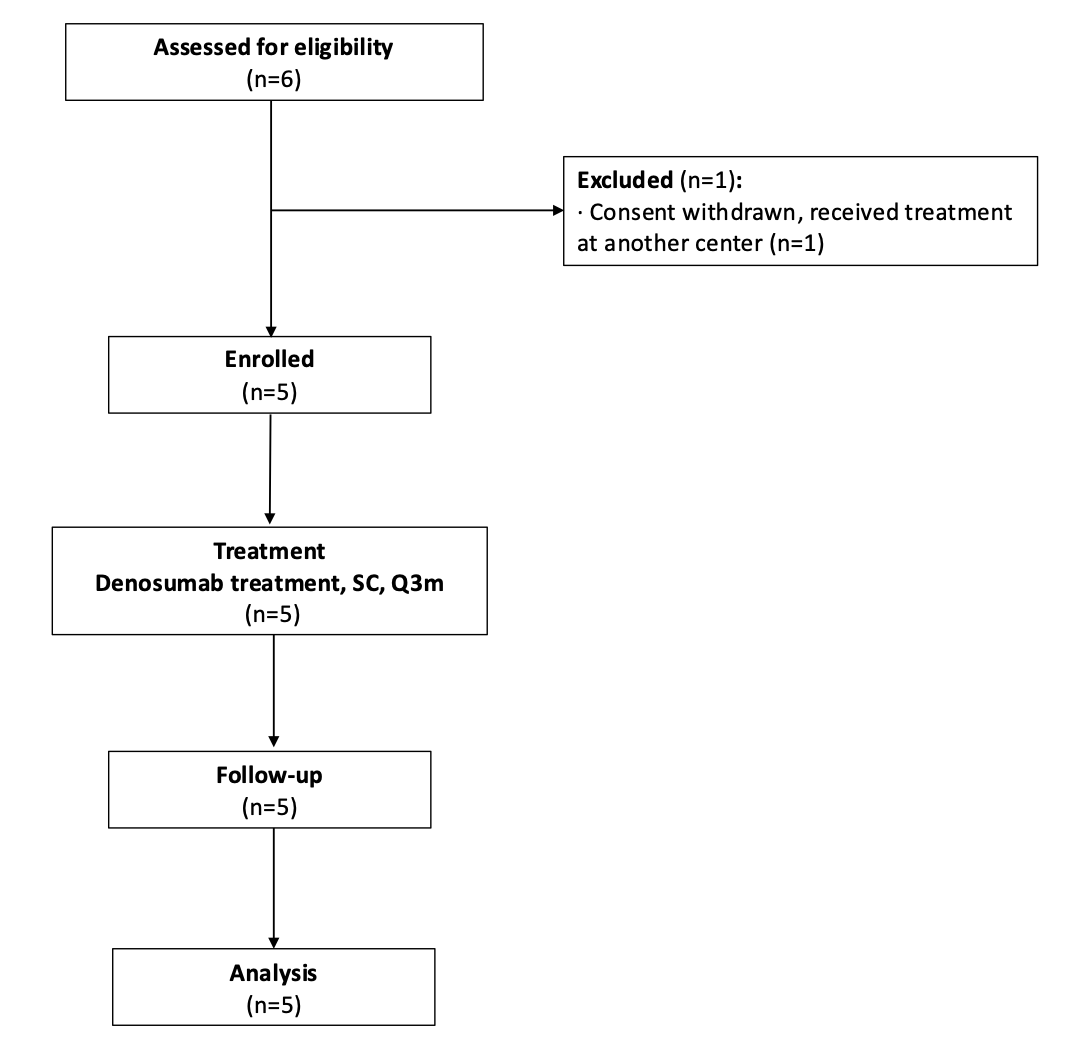


**Supplementary figure 2.** Participant flowchart of the research


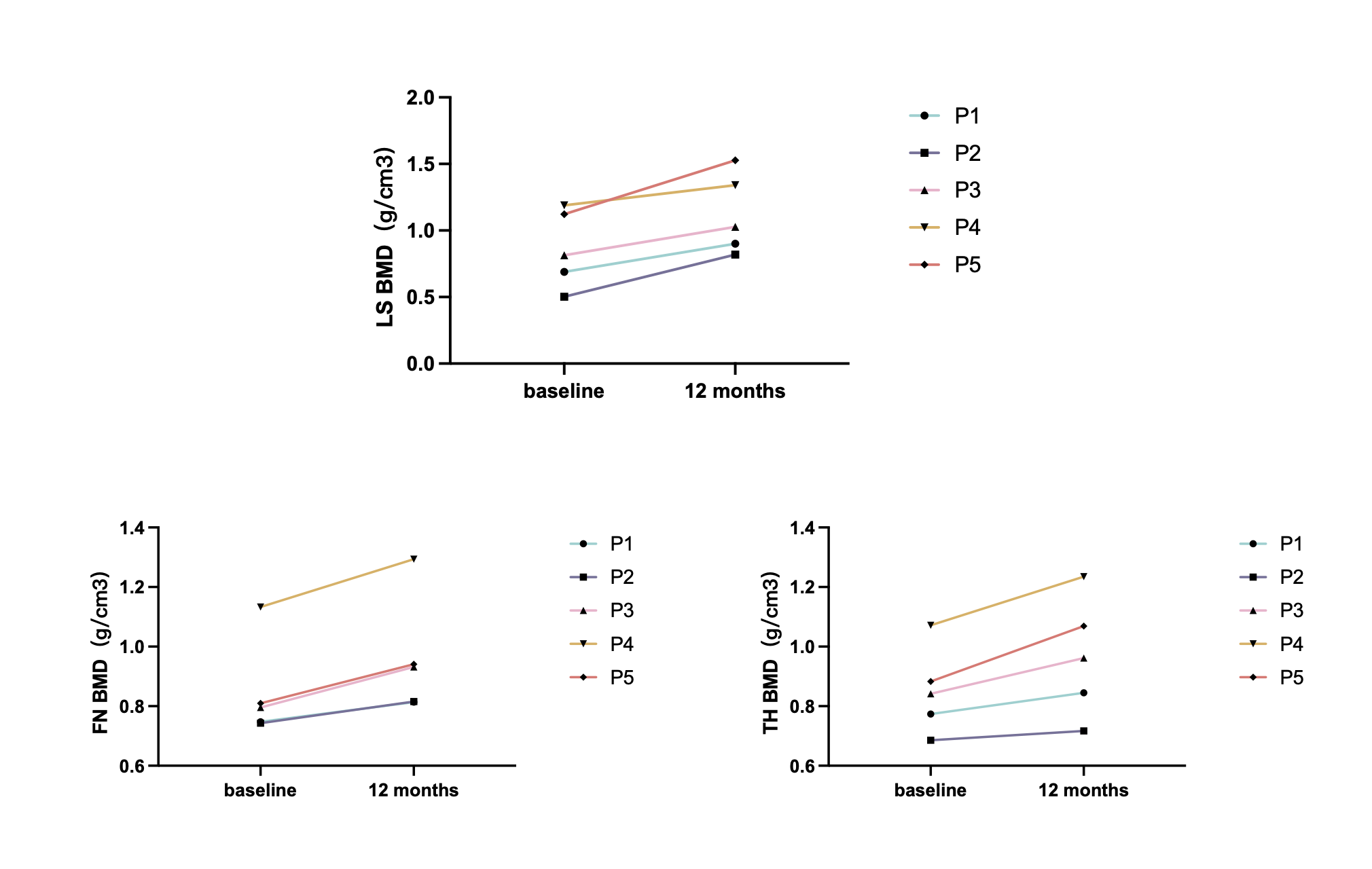


**Supplementary figure 3. trends of bone mass accrual in all patients during follow-up**

**
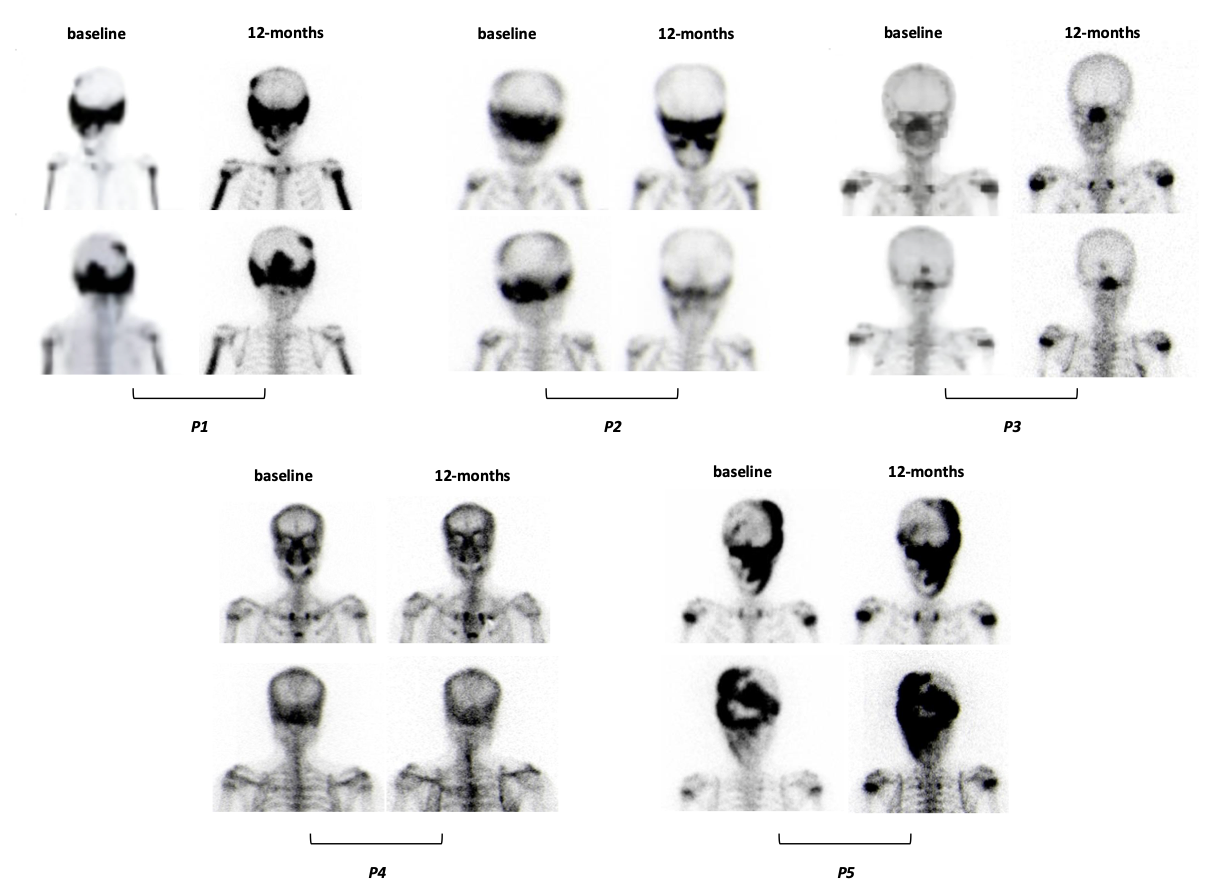
**

**Supplementary figure 4.** trends in changes of cranio-facial radioactivity uptake in 99mTc-MDP bone scintigraphy during follow-up.

**Supplementary table1.** BTMs levels of 5 patients during follow-up

|  | **P1** | | | **P2** | | | **P3** | | | **P4** | | | **P5** | | |
| --- | --- | --- | --- | --- | --- | --- | --- | --- | --- | --- | --- | --- | --- | --- | --- |
|  | ALP  (U/L) | β-CTX  (ng/mL) | P1NP  (ng/mL) | ALP  (U/L) | β-CTX  (ng/mL) | P1NP  (ng/mL) | ALP  (U/L) | β-CTX  (ng/mL) | P1NP  (ng/mL) | ALP  (U/L) | β-CTX  (ng/mL) | P1NP  (ng/mL) | ALP  (U/L) | β-CTX  (ng/mL) | P1NP  (ng/mL) |
| **Baseline** | 789 | 3.70 | >2400 | 731 | 1.99 | 1329 | 585 | 3.59 | 1298 | 203 | 1.19 | 260 | 876 | 5.13 | 1377 |
| **3 months** | 925 | 1.97 | 1847 | 487 | 1.05 | 533 | 213 | 2.55 | 877 | 163 | 0.56 | 194 | 766 | 2.44 | 1144 |
| **6 months** | 697 | 1.05 | 1683 | 245 | 0.26 | 114 | 329 | 2.53 | 455 | 127 | 0.53 | 111 | 432 | 1.94 | 815 |
| **9 months** | 424 | 1.65 | 1403 | 289 | 0.24 | 128 | 278 | 0.36 | 162 | 85 | 0.33 | 55 | 368 | 2.27 | 909 |
| **12 months** | 260 | 1.49 | 581 | 263 | 0.35 | 175 | 228 | 0.31 | 293 | 69 | 0.28 | 48 | 499 | 1.35 | 608 |
